# Energy limitation of cyanophage development: implications for marine carbon cycling

**DOI:** 10.1038/s41396-017-0043-3

**Published:** 2018-01-29

**Authors:** Richard J. Puxty, David J. Evans, Andrew D. Millard, David J. Scanlan

**Affiliations:** 1 School of Life Sciences, University of Warwick, Coventry, West Midlands CV4 7AL UK; 2 School of Biology and BSRC, Biomolecular Sciences Building, North Haugh, St Andrews, KY16 9AJ UK; 3 Department of Infection, Immunity and Inflammation, University of Leicester, Leicester, LE1 9HNL UK

## Abstract

Marine cyanobacteria are responsible for ~25% of the fixed carbon that enters the ocean biosphere. It is thought that abundant co-occurring viruses play an important role in regulating population dynamics of cyanobacteria and thus the cycling of carbon in the oceans. Despite this, little is known about how viral infections ‘play-out’ in the environment, particularly whether infections are resource or energy limited. Photoautotrophic organisms represent an ideal model to test this since available energy is modulated by the incoming light intensity through photophosphorylation. Therefore, we exploited phototrophy of the environmentally relevant marine cyanobacterium *Synechococcus* and monitored growth of a cyanobacterial virus (cyanophage). We found that light intensity has a marked effect on cyanophage infection dynamics, but that this is not manifest by a change in DNA synthesis. Instead, cyanophage development appears energy limited for the synthesis of proteins required during late infection. We posit that acquisition of auxiliary metabolic genes (AMGs) involved in light-dependent photosynthetic reactions acts to overcome this limitation. We show that cyanophages actively modulate expression of these AMGs in response to light intensity and provide evidence that such regulation may be facilitated by a novel mechanism involving light-dependent splicing of a group I intron in a photosynthetic AMG. Altogether, our data offers a mechanistic link between diurnal changes in irradiance and observed community level responses in metabolism, i.e., through an irradiance-dependent, viral-induced release of dissolved organic matter (DOM).

## Introduction

Understanding the response of the biosphere to climate change requires a detailed knowledge of the biological transformation of carbon on Earth. It has become evident that the ocean represents an important sink for atmospheric carbon dioxide (CO_2_) [1]. Just two genera of picophytoplankton dominate open ocean regimes, *Prochlorococcus* and *Synechococcus* [2–4]. Altogether, these closely related genera are responsible for ~25% of oceanic CO_2_ fixation [2]. These organisms have become a model for studying the flow of carbon from CO_2_ to the microbial loop as well as the functioning of planktonic marine communities generally [5, 6]. However, biotic loss rates (antagonistic interactions, grazing, viral lysis) of *Prochlorococcus* and *Synechococcus* remain poorly understood. In the case of viruses, this view is nuanced by the discovery of viral-encoded genes that may act to maintain photosynthesis during infection [7, 8], such that despite the ultimate loss of fixed carbon to dissolved organic matter through lysis, CO_2_ fixation may be maintained transiently during relatively long viral latent periods. Recently, it has been shown that in fact cyanophage shut-down CO_2_ infection early during infection, yet maintain the photosynthetic light reactions [9]. The prevailing view is that cyanophage use excess ATP and reductant from photophosphorylation to fuel DNA replication while inhibiting a costly Calvin Benson cycle [10, 11]. Therefore, we speculated that DNA replication was the limiting factor to cyanophage morphogenesis and that increases in the rate of photophosphorylation resulting from increased irradiance would dramatically alter phage DNA replication kinetics and the resulting productivity of the phage. Such changes would have important consequences for our understanding of viral-induced lysis pressure of open ocean communities given the wide range in light flux in situ.

## Results

### Light intensity modules infection kinetics and photophysiology

We hypothesised that shifting infected *Synechococcus* cells to high light (HL) would provide increased energy for phage replication and as such, we would observe an increase in cyanophage DNA replication rate. To test this idea, we conducted light-shift experiments with *Synechococcus* sp. WH7803, infected with cyanophage S-PM2d [12], and monitored phage development. We conducted qPCR assays quantifying copies of the phage chromosome, from intracellular and extracellular fractions to measure the rate of DNA synthesis inside cells and to determine latent periods and burst sizes

**Fig. 1:**
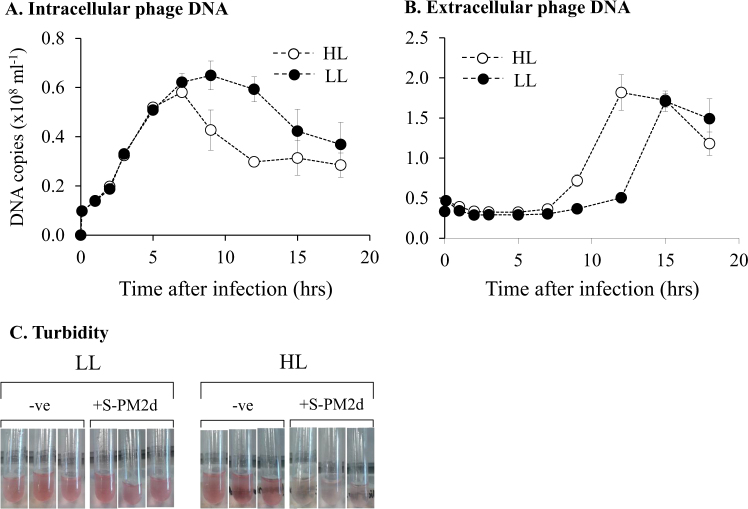
: The effect of light intensity on the kinetics of cyanophage development. **a** qPCR quantification of cyanophage genome copies from the intracellular fraction over the infection period. **b** qPCR quantification of cyanophage genome copies from the extracellular fraction over the infection period. **c** Photographs of culture turbidity taken 10 h after infection

**Fig. 2:**
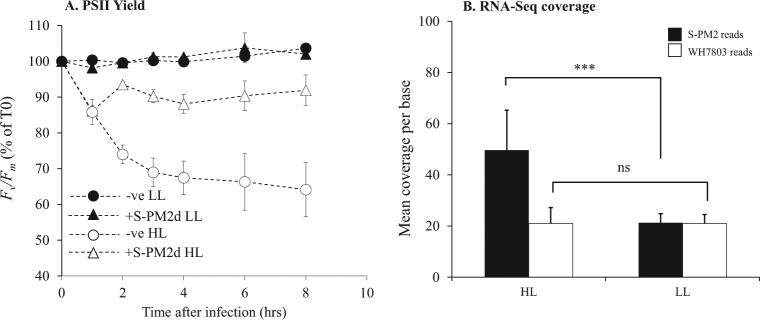
The effect of light intensity on *Synechococcus* photophysiology and cyanophage gene expression. **a** Maximum quantum yield of PSII photochemistry. Unfilled circles (uninfected) and triangles (infected) are samples subjected to the HL treatment and filled circles and triangles are LL. Samples are normalised to time 0. For raw data see Figure S4. **b** Mean per base coverage of the transcriptome across the *Synechococcus* host and cyanophage S-PM2d genome. Filled bars are S-PM2d reads and unfilled bars are *Synechococcus* sp. WH7803 reads. ***p < 0.001, NS not significant. Error bars represent SD from the mean

We observed a major difference in cyanophage development in HL compared with LL (Fig. 1). Under HL the latent period was reduced by 5 h (40% decrease in length), as evidenced both by an earlier reduction in copies of intracellular DNA (Fig. 1a), and an earlier increase in extracellular DNA copy number (Fig. 1b). This early burst under HL conditions was also evident from monitoring culture turbidity (Fig. 1c). In comparison, there was no significant difference in burst size between the different light treatments (Fig. 1b). Surprisingly, and contradicting our proposed hypothesis, there was no difference in the timing, length or rate of phage DNA synthesis between the two conditions (Fig. 1a). We had postulated that increased photochemically driven ATP synthesis under HL would supply more energy and resources to fuel phage DNA replication. Indeed, this idea has been implicated [13] and explicitly modelled [14, 15] as explaining cyanophage acquisition of photosynthesis genes, especially given that other stresses such as phosphate limitation show marked effects on cyanophage DNA synthesis [16]. Our hypothesis relies on the assumption that photochemistry is maintained such that excess absorbed photons can be used to generate a transmembrane potential for ATP synthesis. Therefore, we tested this by measuring photosystem II (PSII) maximum quantum yield (*F*_v_*/F*_m_) during the experiment. This assay measures the differences in the chlorophyll ‘fluorescent transient’ from PSII. As dark-adapted reaction centres become ‘closed’ by the reduction of the primary electron acceptor, there is a concomitant increase in chlorophyll fluorescence. Therefore, the *F*_v_*/F*_m_ parameter is proportional to the number of open reaction centres at a given time [17]. Our measurements of PSII photochemical yield showed no change under LL conditions in infected or uninfected cells (Fig. 2a). In comparison, there was a rapid decline in *F*_v_*/F*_m_ in uninfected cells at HL indicative of photodamage to the PSII reaction centre [18]. After 8 h at HL, *F*_v_*/F*_m_ in uninfected cells stabilises at approximately 65% of the level of LL cultures. Similarly, under HL conditions there was an initial decline in *F*_v_*/F*_m_ in infected cells, at a similar rate to uninfected cells (Fig. 2a). However, there was then an increase in *F*_v_*/F*_m_ 2 h after infection to ~90% of the level at LL where it remained for the rest of the experiment. This observation suggests that not only are PSII reaction centres active in HL infected cells but also either their rate of damage is reduced or the turnover of PSII is much greater to support stable photochemistry at this irradiance.

The maintenance of PSII photochemistry under increased irradiance suggests more energy was available in these cells through photophosphorylation [19]. Yet despite this, we observed no detectable change in the rate, timing or absolute amount of phage DNA synthesis (Fig. 1a, b). We did observe a reduction in the latent period however, and thus we speculate that there may be some temporal control of lysis in response to light. Indeed, bacteriophages have been shown to possess sophisticated mechanisms to delay lysis in response to potential stresses encountered during the infection process [20].

### Cyanophage transcriptional response to high light conditions

**Fig. 3:**
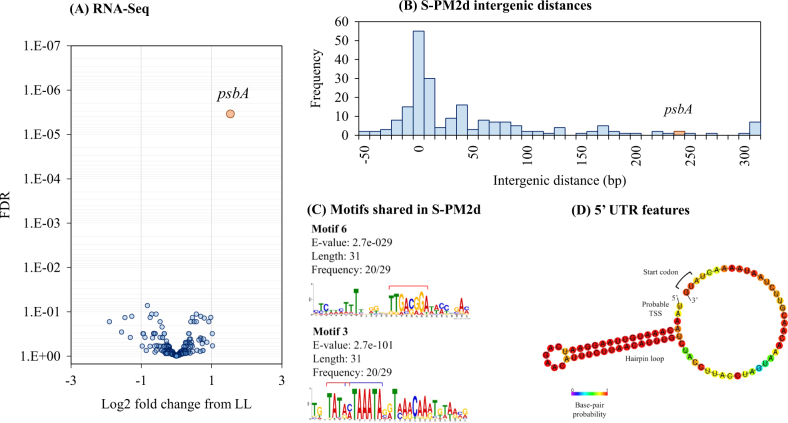
The effect of light intensity on global gene expression. **a** A volcano plot showing the log_2_ fold change of gene expression relative to the LL treatment. The y-axis shows the false discovery rate corrected *p*-value calculated using edgeR. The orange circle shows statistically significant differential gene expression by edgeR. **b** Histogram of intergenic sequence lengths across the cyanophage S-PM2d genome. The bin containing *psbA* is shown in orange. **c** DNA sequence motifs found upstream of cyanomyoviral *psbAs*. Red bars indicate −35 and −10 elements of the σ^70^ binding sites and the blue bar shows the late σ factor Gp55-binding site. **d** Predicted hairpin loop folding structure of the S-PM2d *psbA* 5′-UTR

To decipher potential regulatory mechanisms occurring at the transcriptional level under HL compared to LL conditions, we conducted RNA-Seq analysis of transcriptomes from the same experiment. RNA was extracted from triplicate cultures at 1, 3, 6, and 9 h after infection in HL and LL infected cells and samples from each light treatment pooled in equimolar quantities to assess total changes in expression. Reads were mapped to both the S-PM2d and *Synechococcus* sp. WH7803 reference genomes and mean per base coverage estimated. There was a significant increase in coverage of the phage transcriptome under HL compared to LL conditions (2.34-fold increase, *t*_3_ = 3.98, p = 0.014), which was not apparent in the host (Fig. 2b). This was despite a fraction of the cells undergoing lysis at 9 h in HL compared with no lysis under LL conditions (Fig. 1a, b). This suggests a generalised increased rate of phage transcription at HL despite no change in DNA replication. We used differential expression tests to find light-responsive genes in the cyanophage genome. To our surprise, only one cyanophage gene was proportionately differentially expressed under HL, compared to LL, conditions. This was the photosynthetic AMG *psbA* encoding the D1 polypeptide located at the core of the PSII reaction centre (Fig. 3a).

The cyanophage-encoded *psbA* is a ‘nearly core’ gene in cyanomyoviruses [21], the result of repeated acquisition events from their immediate hosts [22]. It is possible, therefore, that phage acquisition of host genes by recombination has also co-opted the same light-dependent regulatory features of *psbA*. Indeed, other cyanophages have been shown to regulate expression of phosphorus scavenging AMGs in response to phosphate limitation though exploitation of the host’s own PhoBR two-component system [16]. Cyanobacteria, including marine *Synechococcus*, display light-dependent transcription of *psbA*s [18, 23]. In model cyanobacteria, several factors influence the light-dependent accumulation of *psbA* transcripts, including alternative sigma factors [24–28] and DNA tertiary structure [29]. Moreover, D1 degradation products directly bind upstream of *psbA* sequences, such that light-induced damage could directly influence *psbA* transcription in a positive feedback manner [30]. We could not identify any conserved motifs in the upstream region of S-PM2d *psbA* that are shared with their *Synechococcus* hosts, perhaps suggesting divergent regulation strategies between the host and phage versions.

Furthermore, S-PM2d has an uncharacteristically long intergenic sequence upstream of *psbA* (232 bp, compared with a median of 6 bp across the genome; Fig. 3b). This difference is also true for other cyanomyoviruses where the *psbA* upstream regions vary between 125-453 bp. Therefore, we also sought to discover DNA sequence motifs shared amongst cyanomyoviruses that may be involved in regulation of *psbA*. We discovered six high confidence motifs (Fig. S1), of which two were present in S-PM2d (Fig. 3c). These two motifs contain the −35 (motif 6) and −10 (motif 3) elements of the σ^70^ transcription factor binding sites typical in T4-like early phage genes [31]. In addition, motif 3 contains the binding site for the T4-like late gene sigma factor Gp55 [32] (Fig. 3c). Thus, S-PM2d retains motifs required for coordinated expression of *psbA* in early and late infection.

However, the S-PM2d *psbA* region contains a novel 3′ antisense ncRNA, *cfrI* which is known to be expressed [33], as well as a previously unrecognised 36 bp inverted repeat producing a probable RNA hairpin loop in the 5′ untranslated region (5′-UTR; Fig. 3d). Structured 5′-UTRs are well known to affect mRNA stability in bacteria and bacteriophage [34] as are *cis*-encoded antisense RNAs [35], especially for cyanobacterial *psbAs* [36]. These features may therefore contribute to regulation of the cyanophage *psbA* in response to light.

In yet another twist, the S-PM2d *psbA* encodes a group I self-splicing intron within its *psbA* transcript, a feature found to be widespread in cyanophage *psbA* genes from marine metagenomic libraries [33, 37]. The intron interrupts the recognition sequence of a downstream homing endonuclease F-CphI therefore providing self-immunity while allowing the endonuclease to spread amongst intron-less alleles [38]. The intron contains several in-frame stop codons. Thus, it is unlikely that an unspliced transcript encodes a functional D1 polypeptide [33]. Nevertheless, the intron splices efficiently during infection [33].

**Fig. 4:**
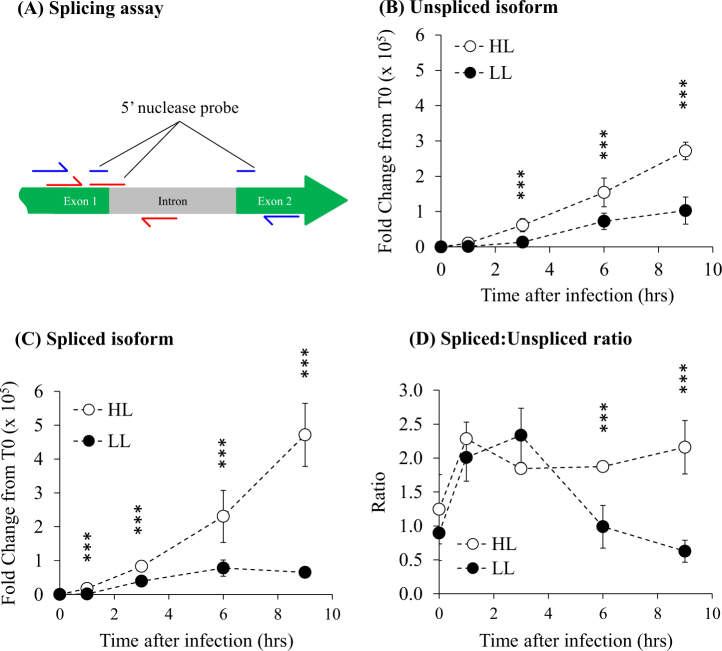
Light-dependent splicing of the S-PM2d *psbA* intron. **a** Schematic of the splicing assay. The blue colour symbolises the primer/probe assay to detect the spliced transcript while red shows the primer/probe assay used to detect the unspliced transcript. **b**–**c** Relative expression of the unspliced isoform and spliced isoform, respectively, over the time-course of infection at HL and LL. **d** The ratio of spliced:unspliced isoforms over the time-course. Error bars represent the standard deviation of the mean. ***p < 0.001 as calculated using REST analysis

To understand whether intron splicing played a role in light-dependent *psbA* expression and indeed to validate our RNA-Seq data we conducted reverse transcriptase quantitative PCR (RT–qPCR) of the cyanophage *psbA* mRNA. We designed a splicing assay to detect relative expression of both isoforms of the transcript (Fig. 4a). We employed a 5′ nuclease qPCR assay which gives specificity to each transcript isoform using a 25–30 bp fluor-quencher linked probe that is either internal to the intron (unspliced isoform detection), or that bridges the exon 1/2 junction (spliced isoform detection). The reverse primer is also located in either the intron (unspliced isoform detection) or on exon 2 (spliced isoform detection) and is specific for cloned copies of each isoform.

The RT–qPCR data validates that obtained from RNA-Seq, showing increased expression of both spliced and unspliced isoforms at HL (Fig. 4b, c). This increase is particularly pronounced in the spliced isoform during late infection, where we observed a 10.6-fold increase at HL compared with LL (Fig. 4c), compared with only a 3.37 fold increase of the unspliced isoform. Importantly, our assay allows determination of the spliced: unspliced isoform ratio (Fig. 4d), which shows that throughout early infection up to 4 h, the phage maintains a ~2-fold increase in the amount of the spliced isoform (Fig. 4d). At HL this is maintained throughout infection, whereas in LL we observed a rapid decline to approximately equimolar amounts by 6 h. Thus, the splicing of the intron appears to be responsive to light intensity. To our knowledge, this is the first report of a differentially spliced intron in a bacteriophage, though interestingly the eukaryotic alga *Chlamydomonas reinhardtii*, contains four group I introns in its *psbA* gene [39], which are known to be differentially spliced in response to light [40]. Since unspliced transcripts likely do not encode functional polypeptides, we suggest that differential splicing of the *psbA* mRNA represents a mechanism of gene regulation, potentially in coordination with the antisense ncRNA *cfrI* and/or the structured 5′-UTR affecting mRNA stability.

### Differential expression of host genes reinforce translational bottlenecks

In addition to the differential expression of cyanophage genes, we also analysed light-responsive *Synechococcus* host genes. Of the 39 genes upregulated under high light conditions, 25 (64%) were involved in translation or posttranslational modification, protein turnover and chaperones (Table 1). This included 13/31 and 4/22 proteins forming the 50 S and 30 S subunits of the ribosome, respectively. Moreover, both subunits of the GroEL/GroES complex as well as the DnaJ and DnaK chaperones were upregulated (Table 1). Meanwhile, three of the four copies of the host’s *psbA* gene were also upregulated in high light (Table 1), perhaps complementing phage supplies of *psbA* mRNAs. Downregulated genes mostly included components of the light harvesting apparatus, potential cell motility genes and subunits involved in linear electron transport—most notably photosystem I reaction centre subunits (Table 1).

**Table 1:** Differentially expressed *Synechococcus* host genes between high and low light conditions during infection with cyanophage S-PM2. Positive and negative log2 fold changes indicate up/down regulation respectively with respect to the low light treatment

Locus Tag	Gene Name	Annotation	Functional group	Log2 Fold Change in expression	adj-*p*-value
*Upregulated*					
SYNWH7803_0424	*rplN*	50 S ribosomal protein L14	Translation	1.348	3.41E−02
SYNWH7803_2372	*rplJ*	50 S ribosomal protein L10	Translation	1.246	2.41E−12
SYNWH7803_0434	*rplC*	50 S ribosomal protein L3	Translation	1.240	1.69E−08
SYNWH7803_0431	*rplB*	50 S ribosomal protein L2	Translation	1.209	4.96E−05
SYNWH7803_1329	*rpsB*	30 S ribosomal protein S2	Translation	1.200	9.88E−06
SYNWH7803_0429	*rplV*	50 S ribosomal protein L22	Translation	1.171	1.23E−02
SYNWH7803_0422	*rplE*	50 S ribosomal protein L5	Translation	1.138	8.77E−05
SYNWH7803_0427	*rplP*	50 S ribosomal protein L16	Translation	1.102	1.23E−02
SYNWH7803_2374	*rplK*	50 S ribosomal protein L11	Translation	1.089	3.85E−04
SYNWH7803_0591	*rpsD*	30 S ribosomal protein S4	Translation	1.072	3.53E−03
SYNWH7803_0433	*rplD*	50 S ribosomal protein L4	Translation	1.033	5.82E−03
SYNWH7803_0428	*rpsC*	30 S ribosomal protein S3	Translation	1.033	2.68E−03
SYNWH7803_0418	*rpsE*	30 S ribosomal protein S5	Translation	1.003	5.55E−03
SYNWH7803_0423	*rplX*	50 S ribosomal protein L24	Translation	1.001	1.76E−03
SYNWH7803_0420	*rplF*	50 S ribosomal protein L6	Translation	0.972	1.66E−03
SYNWH7803_2371	*rplL*	50 S ribosomal protein L7/L12	Translation	0.924	8.30E−06
SYNWH7803_2373	*rplA*	50 S ribosomal protein L1	Translation	0.805	1.56E−02
SYNWH7803_1863	*groEL*	60 kDa chaperonin 2	Posttranslational modification, protein turnover, chaperones	1.520	7.06E−15
SYNWH7803_1118	*ahpC*	Peroxiredoxin, AhpC/TSA family	Posttranslational modification, protein turnover, chaperones	1.315	3.65E−05
SYNWH7803_1998	*groEL*	60 kDa chaperonin 1	Posttranslational modification, protein turnover, chaperones	1.171	5.63E−12
SYNWH7803_0023	SynWH7803_0023	DnaJ-class molecular chaperone	Posttranslational modification, protein turnover, chaperones	1.043	3.61E−02
SYNWH7803_2514	*dnaK*	Molecular chaperone DnaK	Posttranslational modification, protein turnover, chaperones	0.849	5.15E−07
SYNWH7803_0016	*msrB*	Peptide methionine sulfoxide reductase	Posttranslational modification, protein turnover, chaperones	0.758	1.18E−03
SYNWH7803_1999	*groES*	10 kDa chaperonin	Posttranslational modification, protein turnover, chaperones	0.743	1.82E−02
SYNWH7803_1555	*pepN*	Aminopeptidase N	Posttranslational modification, protein turnover, chaperones	0.603	2.50E−02
SYNWH7803_0790	*psbA*	Photosystem II protein D1	Photosynthesis	1.795	8.48E−04
SYNWH7803_2084	*psbA*	Photosystem II protein D1	Photosynthesis	1.695	5.50E−04
SYNWH7803_0366	*psbA*	Photosystem II protein D1	Photosynthesis	1.578	5.53E−04
SYNWH7803_1861	*fabG*	3-oxoacyl-[acyl-carrier-protein] reductase	Fatty acid biosynthesis	1.023	4.52E−02
SYNWH7803_1870	SynWH7803_1870	Uncharacterised protein required for cytochrome oxidase assembly	Energy production and conversion	1.599	3.16E−03
SYNWH7803_2017	*atpA*	ATP synthase alpha chain	Energy production and conversion	0.718	1.21E−02
SYNWH7803_2495	*cynS*	Cyanate lyase	Cyanate metabolism	0.616	3.14E−03
SYNWH7803_0169	*ahcY*	Adenosylhomocysteinase	Coenzyme transport and metabolism	1.219	1.18E−03
SYNWH7803_2069	SynWH7803_2069	Carbohydrate-binding protein; modular; contains a central CBM2 module	Carbohydrate transport and metabolism	1.998	4.00E−11
SYNWH7803_1930	*glgX*	Glycogen debranching enzyme	Carbohydrate transport and metabolism	1.814	1.08E−03
SYNWH7803_1423	SynWH7803_1423	Conserved hypothetical protein		1.490	4.99E−02
SYNWH7803_2070	SynWH7803_2070	Two-component system response regulator		1.119	5.22E−03
SYNWH7803_0046	SynWH7803_0046	DHSS soluble hydrogenase, small subunit		0.809	4.27E−02
SYNWH7803_1728	SynWH7803_1728	Integral membrane protein		0.586	1.82E−02
*Downregulated*					
SYNWH7803_0611	*rpoD*	RNA polymerase sigma factor RpoD, sigma-70 family	Transcription	−0.746	1.22E−04
SYNWH7803_2501	*rpoD*	Alternative RNA polymerase sigma factor, sigma-70 family	Transcription	−1.037	4.76E−04
SYNWH7803_1592	*trxA*	Thioredoxin	Redox homoeostasis	−0.882	7.75E−04
SYNWH7803_2137	SynWH7803_2137	Predicted ATPase with chaperone activity	Posttranslational modification, protein turnover, chaperones	−1.957	9.76E−03
SYNWH7803_0353	*psbO*	Photosystem II manganese-stabilising protein PsbO	Photosynthesis	−0.500	4.78E−02
SYNWH7803_0984	*petJ*	Cytochrome C6	Photosynthesis	−0.739	4.36E−02
SYNWH7803_0252	*ndhC*	NAD(P)H-quinone oxidoreductase chain 3	Photosynthesis	−0.768	3.63E−02
SYNWH7803_0391	*psaA*	Photosystem I P700 chlorophyll A apoprotein A1	Photosynthesis	−0.829	1.46E−06
SYNWH7803_0534	*petD*	Cytochrome b6-f complex subunit 4	Photosynthesis	−0.869	1.18E−03
SYNWH7803_1224	*psaK*	Photosystem I reaction centre subunit X, PsaK	Photosynthesis	−0.983	1.45E−03
SYNWH7803_0396	*psaL*	Photosystem I reaction centre subunit XI	Photosynthesis	−1.001	3.18E−04
SYNWH7803_0392	*psaB*	Photosystem I P700 chlorophyll A apoprotein A2	Photosynthesis	−1.078	2.41E−12
SYNWH7803_0502	*mpeD*	Phycobilisome linker polypeptide, C-phycoerythrin-associated	Light Harvesting	−0.543	2.88E−02
SYNWH7803_0500	SynWH7803_0500	Conserved hypothetical protein in phycobilisome rod gene region	Light Harvesting	−0.884	6.12E−04
SYNWH7803_0493	*mpeB*	C-phycoerythrin class II beta chain	Light Harvesting	−1.034	7.27E−10
SYNWH7803_0501	*cpeE*	Phycobilisome linker polypeptide, C-phycoerythrin class I-associated	Light Harvesting	−1.042	4.63E−05
SYNWH7803_0492	*mpeA*	C-phycoerythrin class II alpha chain	Light Harvesting	−1.312	1.05E−09
SYNWH7803_0670	*chlL*	Protoporphyrin IX Mg-chelatase subunit ChlD	Chlorophyll biosynthesis	−1.493	5.53E−15
SYNWH7803_0029	*gap1*	Glyceraldehyde-3-phosphate dehydrogenase	Central carbon metabolism	−0.944	5.50E−04
SYNWH7803_0543	SynWH7803_0543	LysM-repeat protein	Cell wall/membrane biogenesis	−0.533	9.89E−03
SYNWH7803_1746	*murA*	UDP-N-acetylglucosamine 1-carboxyvinyltransferase	Cell wall/membrane biogenesis	−1.010	1.37E−02
SYNWH7803_1841	*pilT*	Twitching motility protein	Cell motility	−1.435	3.85E−14
SYNWH7803_1798	SynWH7803_1798	Uncharacterized conserved secreted protein, pili subunit superfamily	Cell motility	−1.524	4.63E−08
SYNWH7803_1796	SynWH7803_1796	Uncharacterized conserved secreted protein, pili subunit superfamily	Cell motility	−1.540	1.21E−12
SYNWH7803_1795	SynWH7803_1795	Uncharacterized conserved secreted protein, pili subunit superfamily	Cell motility	−1.658	9.13E−29
SYNWH7803_0756	SynWH7803_0756	Alpha-glycosidase of family GH13; possible 1, 4-alpha-glucan branching enzyme	Carbohydrate transport and metabolism	−1.070	3.15E−03
SYNWH7803_1937	SynWH7803_1937	Conserved hypothetical protein		−0.547	1.83E−02
SYNWH7803_0291	SynWH7803_0291	Conserved hypothetical protein		−0.657	5.56E−03
SYNWH7803_1897	SynWH7803_1897	Uncharacterized conserved secreted protein		−0.659	4.11E−02
SYNWH7803_0551	SynWH7803_0551	Hypothetical protein		−0.659	1.18E−02
SYNWH7803_2356	SynWH7803_2356	Uncharacterized conserved secreted protein		−0.750	1.18E−03
SYNWH7803_0849	SynWH7803_0849	Conserved hypothetical protein		−0.864	1.59E−02
SYNWH7803_0308	SynWH7803_0308	Secreted protein with pentapeptide repeats		−0.912	2.42E−03
SYNWH7803_2182	SynWH7803_2182	Conserved hypothetical protein		−0.922	1.77E−05
SYNWH7803_0069	SynWH7803_0069	Conserved hypothetical protein		−1.122	7.64E−04
SYNWH7803_2423	SynWH7803_2423	Conserved hypothetical protein		−1.160	2.20E−04
SYNWH7803_1348	SynWH7803_1348	Hypothetical protein		−1.325	9.08E−04
SYNWH7803_0705	SynWH7803_0705	Hypothetical protein		−1.393	3.38E−06
SYNWH7803_0839	SynWH7803_0839	Hypothetical protein		−1.394	1.56E−02
SYNWH7803_1797	SynWH7803_1797	Uncharacterized conserved secreted protein		−1.509	2.30E−03
SYNWH7803_2085	SynWH7803_2085	Uncharacterized conserved secreted protein		−1.612	2.27E−08
SYNWH7803_1502	SynWH7803_1502	Conserved hypothetical membrane protein		−1.629	9.89E−03
SYNWH7803_1800	SynWH7803_1800	Uncharacterized conserved membrane protein		−1.655	4.20E−17
SYNWH7803_0463	SynWH7803_0463	Uncharacterized conserved membrane protein		−1.757	3.86E−04
SYNWH7803_0464	*pex*	Possible Pex protein (Period-extender gene product)		−2.178	9.21E−12
SYNWH7803_0940	SynWH7803_0940	Conserved hypothetical protein		−2.185	3.01E−14
SYNWH7803_2186	SynWH7803_2186	Conserved hypothetical protein		−2.274	7.21E−20
SYNWH7803_0934	SynWH7803_0934	Hypothetical protein		−3.056	2.56E−88

## Discussion

**Fig. 5:**
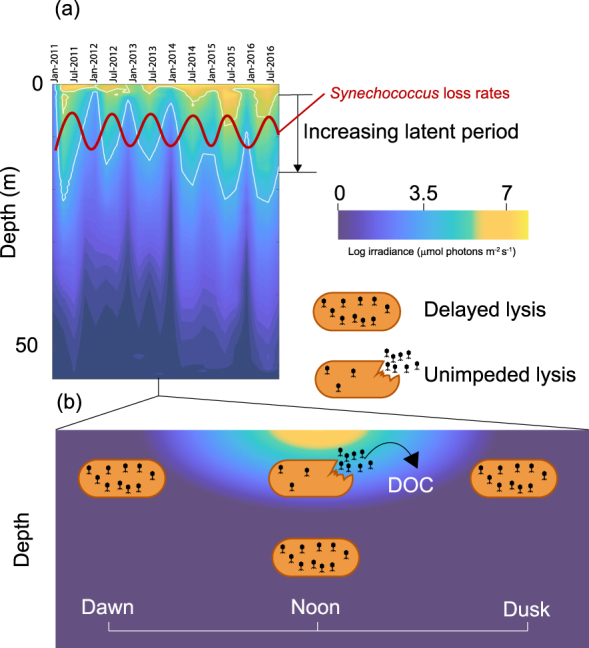
The ecological significance of light-dependent lysis delay. **a** Seasonal and inter-annual changes in irradiance with depth at a coastal observatory (the data shown here is from station L4 in the Western English Channel, the site of isolation of cyanophage S-PM2d). White lines correspond to the contours of the irradiances used in this study. The red line shows seasonal trends in *Synechococcus* loss rates [51]. **b** Diurnal changes in irradiance. Delayed lysis at low light results in a reduction in the rate of DOC release. Lysis at increased irradiances increases the rate of DOC release through the viral shunt

Altogether, our data show that light intensity plays a key role in modulating cyanophage development in their *Synechococcus* host. In contrast to our prediction, there was no change in the rate or amount of DNA replication. Instead, increased irradiance induced an early burst phenotype. Other than *psbA*, which was overexpressed under HL conditions, there was no change detectable in cyanophage gene expression. The encoded D1 protein binds all the proteins and cofactors required for charge separation leading to the oxidation of water [41]. The proximity to the charge separation event means D1 is particularly susceptible to damage and as such is rapidly turned over at HL [42]. Thus, upregulation of cyanophage *psbA* under HL conditions may increase the rate of delivery of D1 to PSII, restoring activity of the reaction centre, which in turn is manifest as the observed increase in PSII photochemistry (Fig. 2a). The resulting effect is an increase in the rate of photophosphorylation and more energy for cyanophage development. Taken together, and with no obvious change in expression of other cyanophage genes, we suggest that it is this increase in energy that supports an earlier burst under HL conditions, and therefore that translation is the rate limiting step for cyanophage development during late infection. In this respect, we calculated the energy requirement of cyanophage DNA replication assuming a burst size of 10. This calculation is based on known pathways of pyrimidine and purine deoxyribonucleotide biosynthesis in *Synechococcus* (Fig. S2). In total, we estimate a requirement of 2.2 × 10^−17^ mol ATP per infection (Supplementary Information). This assumes that all dNTPs are synthesised de novo, which is therefore an overestimate due to the several pathways predicted for nucleotide salvage in the S-PM2d genome [43]. In comparison, we computed the energy cost of production of just the detected structural proteins of cyanophage S-PM2 [44]. This includes both the biosynthesis (7.4 × 10^−19^ mol ATP per infection) but also crucially the polymerisation of amino acids (4.4 × 10^−17^ mol ATP per infection) (Supplementary Information). Thus, there is a 2-fold increase in energy required for translation of just the known structural proteins that form the phage particle compared with that of DNA synthesis. This must underestimate the overall increase in energy required as it does not include structural proteins that have yet to be identified, or the non-structural proteins critical for particle assembly, genome replication or subversion of host metabolism. Clearly then, translation represents the major energetic cost for cyanophage development. This conclusion is supported both by recent modelling studies into the energetics of development of coliphage T4 [45] and our own analysis of the host transcriptional response during infection under higher irradiance. The latter specifically showed upregulation of components of the bacterial ribosome as well as several chaperone proteins presumably involved in aiding correct folding of nascent polypeptides. We predict that upregulation of these genes will cause increases in the local concentration of ribosomes which would support an increased translation rate at higher light [46]. Unfortunately, this has largely been overlooked in previous cyanophage work since acquisition of AMGs has been proposed to overcome metabolic bottlenecks to increase cyanophage genome replication [7, 14, 15]. Our data suggests that the energy required for translation during late infection imposes such a metabolic block, and that acquisition and coordinated expression of the cyanophage S-PM2 *psbA* AMG overcomes this energy bottleneck. We predict that energy limitation scales with light intensity such that reduced latent periods would be expected during the day and at shallower depths (Fig. 5). Indeed, cyanophage development has been shown to be inhibited by darkness [7, 47] and infections with cyanosiphoviruses over a simulated diel cycle show that latent period length is correlated with light intensity [48]. Moreover, isolation studies show increased abundance of cyanophages at mid-day, but only at shallower depths with increased irradiance [49]. In addition, recent lagrangian metagenomics sampling of viral communities in waters of the north Pacific subtropical gyre suggest cyanophages exhibit diel rhythmicity, peaking in intracellular abundance between mid-day to early evening [50]. Given that *Synechococcus* and the closely related *Prochlorococcus* are dominant members of the photoautotrophic community in the global ocean, and that natural gradients of light intensity occur with depth, time of day and season, it is likely that energy limitation of cyanophage development would have important implications for the control of the *Synechococcus* community. For instance, it has recently been shown than *Synechococcus* exhibits a regular spring bloom in coastal waters off Woods Hole, USA [51]. This bloom is driven by increases in *Synechococcus* cell division rates in response to warmer water temperature (but importantly not light) and decoupling from loss rates in early spring. It may be that extended viral latent periods caused by light limitation are prominent in early spring. These would cause reduced *Synechococcus* loss rates, contributing to bloom formation. Conversely, the *Synechococcus* loss rate is maximal during mid-summer [51], when increased irradiance may shorten viral latent periods (Fig. 5a). This is particularly relevant in a warming ocean where *Synechococcus* blooms have already been shown to be occurring earlier in the year [51]. The effect of light on latent periods of *Synechococcus* likely also affect the daily and depth-dependent cycling of carbon in the oceans, with diurnal, depth and seasonal variability in the release of dissolved organic carbon (DOC) via the viral shunt (Fig. 5b) [52, 53]. Recently, whole-community metatranscriptomics has revealed reproducible daily trends in the expression of genes involved in oxidative phosphorylation and translation amongst the heterotrophic component of surface ocean communities [54, 55]. Peak expression coincides with daily maxima in irradiance and are subsequent to expression of photosynthetic genes by the phototrophic community members. Therefore, high light-induced lysis by viruses of phototrophs could link these two processes. Future studies should seek to determine the generality of this phenomenon amongst cyanophage and understand the relationship between rates of photophosphorylation and the energy requirement for lysis over a range of irradiances and varying host nutrient status so that such information can be integrated into models of oceanic C cycling.

## Methods

### Infection conditions

*Synechococcus* sp. WH7803 was grown in ASW medium [56] to 1 × 10^8^ cells ml^−^^1^ at 23˚C under continuous illumination of 15 µmol photons m^−2^ s^−^^1^. Cultures were then infected with cyanophage S-PM2d [12] at a VBR of 10 and samples incubated at LL (15 µmol photons m^−2^ s^−1^) for 1 h to allow adsorption. Three biological replicates were then shifted to 210 µmol photons m^−^^2^ s^−^^1^ (HL) while three control cultures were maintained at 15 µmol photons m^−^^2^ s^−^^1^ (LL). The HL treatment was selected based on the ability to induce transient photoinhibition in uninfected cells without being lethal. Photoinhibition was assessed by determining the PSII maximum photochemical yield (*F*_v_*/F*_m_) after treatment with lincomycin following [18]. The rate of photoinhibition for the HL treatment is shown in Fig. S3.

### Assessment of infection dynamics

Infection dynamics were assayed using qPCR to enumerate both intracellular and extracellular cyanophage genome copy number (GCN) [11, 16, 57]. Samples were taken immediately after addition of cyanophage S-PM2d and then at 1, 2, 3, 5, 7, 9, 12, 15 and 18 h. For quantification of extracellular cyanophages 100 µl cell suspension was diluted to 500 µl by addition of ASW medium. This was syringe filtered through a 0.2 µm pore size disposable filter (Minisart, Sartorius, Goettingen, Germany). The filtrate was collected, snap frozen in liquid nitrogen, and then stored at −80 ˚C before quantification. Intracellular cyanophages were quantified by vacuum filtration of infected cells onto a 0.2 µm pore size polycarbonate filter (Isopore, Millipore, Billerica, USA). Filters were washed three times with 1 ml preservation solution (10 mM Tris-HCl, 100 mM EDTA, 500 mM NaCl, pH 8.0) and after their removal from the filtration tower added to a ribolyser tube (Lysing Matrix E, MP Bioproducts, CA, USA) and snap frozen in liquid nitrogen for DNA extraction.

### DNA extraction

DNA extraction was extensively optimised and the following was found to yield optimal results. 650 µl Tris-HCl, pH 8.0 was added to a ribolyser tube (Lysing Matrix E, MP Bioproducts, CA, USA). The tube was then lysed three times for 30 s in a Tissue Lyser (Qiagen, Hilden, Germany) at 30 Hz. In between disruption the tubes were incubated on ice for 1 min. The lysate was subjected to centrifugation for 30 s at 10,000×*g* and 100 µl of the supernatant subsequently frozen at −80 ˚C. Samples were diluted 1/10 and 1 µl was used as the template for qPCR reactions.

### qPCR

qPCR enumeration of cyanophage S-PM2d genome copies was achieved using a PrimeTime 5′ nuclease assay (IDT, Coralville, USA). Primer/probe sequences are shown in the Supplementary Information and were directed towards the *psbA* intron of S-PM2d. 20 µl reaction volumes were used containing 1 × Brilliant III Ultra-Fast qPCR Master Mix (Agilent, CA, USA), 500 nM primers and 250 nM probe, 1 µl template and nuclease free water. Each qPCR plate contained a triplicate dilution series from the initial phage stock from 2.5 × 10^10^ −2.5 × 10^2^ pfu ml^−^^1^. Cycling conditions were 3 min at 95 ˚C, followed by 40 cycles of denaturing for 15 s at 95 ˚C and annealing for 15 s at 60 ˚C.

### Photophysiology

The maximum quantum yield of PSII photochemistry (*F*_v_*/F*_m_) was measured according to [18]. All measurements were made using a pulse amplitude-modulated fluorometer (PhytoPAM, Walz, Effeltrich, Germany). Subsamples (2 ml) were incubated for ~5 min in the dark to completely oxidise the primary electron acceptor Q_A_. 500 µl subsample was added to a PhytoPAM cuvette (Waltz, Effeltrich, Germany) and immediately diluted to 2 ml with ASW medium. A weak modulating light was then applied at 520 nm with intensity of 1 µmol photons m^−^^2^ s^−^^1^ and the basal fluorescence measured (*F*_0_). 3-(3,4-dichlorophenyl)-1,1-dimethylurea (DCMU) was added to the cuvette to a final concentration of 100 µM, and an actinic light was supplied at ~1300 µmol photons m^−^^2^ s^−^^1^. Chlorophyll fluorescence rose rapidly followed by a slower period of increase until saturation. A saturating pulse of ~2600 µmol photons m^−^^2^ s^−^^1^ was then delivered for 200 ms to completely reduce Q_A_. The fluorescence was simultaneously measured (*F*_m_) and *F*_v_*/F*_m_ calculated as *(F*_m_*−F*_0_*)/F*_m_.

### RNA extraction and RNA-Seq library preparation

Total RNA was isolated following Logemann et al. [58]. Briefly, 50 ml of *Synechococcus* sp. WH7803 was centrifuged at 3220 × g at 4°C and flash frozen in liquid N_2_. Pellets were thawed in 1.5 ml Z buffer (8 M guanidinium hydrochloride; 50 mM β-mercaptoethanol; 20 mM EDTA) at room temperature for 30 min. 2 volumes of acidified phenol (pH 4.5) were added and heated to 65˚C for 30 min, followed by the addition of chloroform:isoamyl alcohol for 15 min. The aqueous phase was transferred to separate microcentrifuge tubes. RNA was precipitated by the addition of 1 vol. isopropanol followed by incubation overnight at 4˚C and subsequent centrifugation at 16,060×*g* (Biofuge Pico, Heraeus) at 4 ˚C for 30 min. The pellet was then washed with 70% (v/v) ethanol and further centrifuged for 15 min. Pellets were resuspended in 50 µl H_2_O. DNA was removed using the TURBO DNA-free kit (Ambion/Life Technologies, Carlsbad, USA) following the manufacturer’s instructions. gDNA contamination was tested by PCR using primers phoH_F/phoH_R (Table S1). Any samples that yielded a PCR product were further treated using the TURBO DNA-freeTM kit (Ambion, Forster City, USA).

### Strand-specific library preparation

For each biological replicate, total RNA from time points 1, 3, 6, and 9 h after infection were pooled in equimolar quantities. RNA-Seq libraries were prepared using the ScriptSeq v2.0 kit (Illumina, San Diego, USA) by the Centre for Genomic Research, University of Liverpool and sequenced at the same facility using an Illumina GAIIx platform generating 100 bp paired-end reads. Raw reads have been submitted to ArrayExpress (EMBL-EBI) under accession E-MTAB-5840.

### Read-mapping, read counts and differential expression tests

Paired-end reads were trimmed using Sickle [59] using default settings. Trimmed reads were mapped to the S-PM2d (E.B.I. Accession no. LN828717) and *Synechococcus* sp. WH7803 genome (Acc. No. NC_009481) using Bowtie 2 [60]. Mapping was done in –end-to-end mode using the following parameters: -D 20 -R 3 -N 0 -L 20 -i S,1,0.50. SAM files were converted to BAM files and sorted using SAMtools v0.1.18 [61] and reads counts of genomic features were calculated using BEDtools v2.26.0 [62]. Only reads which overlapped across 10% of the read length into the genomic feature were counted. Differential expression tests were conducted using EdgeR [63] in the Degust online form.

### RT–qPCR

cDNA synthesis was carried out with SuperScript III reverse transcriptase (Life Technologies, Carlsbad, USA) in 20 µl volumes with 2 µg total RNA. Each reaction contained 0.5 mM dNTPs, 2 pM gene-specific primer, 1× SuperScript III buffer, 5 mM DTT and 200U SuperScript III. The RNA, dNTP and primers were mixed and heated to 65˚C for 5 mins before being cooled on ice for 5 mins. SuperScript III, DTT and buffer were then added, mixed, and incubated at 55˚C for 60 mins. The reaction was inactivated by heating to 70 ˚C for 15 mins. qPCR reactions were in 20 µl volumes containing 1× Brilliant III Ultra-Fast qPCR Master Mix (Agilent, Santa Clara, USA), 1× relevant primer/probe assay (500 nM primers and 250 nM probe), 1 µl cDNA and nuclease free water to 20 µl. An Applied Biosystems 7500 Fast Real-Time PCR System (Life Technologies, Carlsbad, USA) was used for quantification. Cycling parameters were 95 ˚C for 1 min followed by 40 cycles of 15 s at 95˚C and 15 s at 60˚C. Raw CT values were exported and analysed using the efficiency corrected ΔΔCT method [64] as computed using the REST software [65]. The calibrator gene was the host 16S rRNA gene when quantifying spliced and unspliced transcripts and the unspliced transcript when calculating the ratio shown in Fig. 4d. Primer sequences and assay validations are in the Supplementary Information. The specificity of the spliced and unspliced *psbAs* were validated using cloned copies of each isoform.

### Motif discovery

Motifs upstream of *psbAs* were detected using MEME [66] using a minimum width of 6 bp and maximum of 50 bp and an eval cutoff of 10^−^^5^.

## Electronic supplementary material

Supplementary Information

Figure S1
