# Supplementary material for: Energy limitation of cyanophage development: implications for marine carbon cycling
Source: ISME J. 2018 Jan 29;12(5):1273–86. doi: 10.1038/s41396-017-0043-3 (PMC5931967; doi:10.1038/s41396-017-0043-3)
Supplement: Supplementary file 1 — Supplementary Information [file 41396_2017_43_MOESM1_ESM.docx]

Table S1: Primer sequences used in this study

| Primer Name | Sequence (5'-3') |
| --- | --- |
| psbA_intF | CTGGTCTGGGTATGGAGGTG |
| psbA_intR | TGTCGGACGCTTATTCCTGT |
| psbA_intProbe | 5Cy5/ACGAGCGTCAAGTTGTGCTTTGCGC/3IAbRQSp |
| psbA_spl_F | CTGGTCTGGGTATGGAGGTG |
| psbA_spl_R | AGGCAACTGGTGTTGCTTCT |
| psbA_spl_probe | 5TET/CACGAGCGTAATGCACACAACTTCCCTCTT/3IABkFQ |
| 16S_F | CCAAGGCATCGATCAGTAGCT |
| 16S_R | CTGGGCCGTGTCTCAGT |
| 16S_probe | CCAGTGTGGCTGATCAT |
| phoH_F | TCTTCTTGGCATCGAACCACT |
| phoH_R | GCGTTGGAAATCTAAGATCCCC |

Table S2: Parameters of validated qPCR assays used in this study

| Parameter | 16S | *psbA*_Unspliced | *psbA_*Spliced |
| --- | --- | --- | --- |
| Amplification Efficiency (E) | 1.06 | 1.12 | 1.13 |
| *R^2^* | 0.9983 | 0.9986 | 0.9981 |
| Dynamic Range (CTs) | 9.115-18.735 | 9.354-35.876 | 9.192-36.019 |


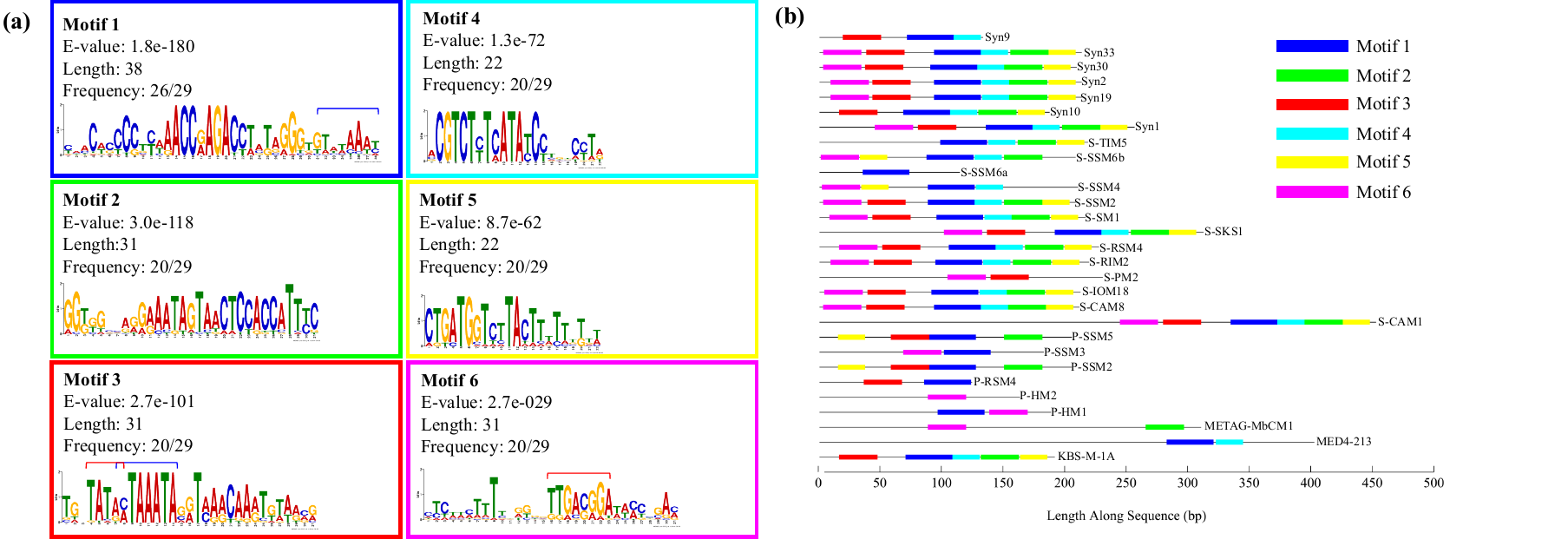


Figure S1: Conserved motifs in upstream regions of cyanophage encoded psbAs. (a) Information content of each motif. The frequency refers to the number of taxa containing each motif. Blue and red lines indicate matches to the T4-like late and early promoters respectively. (b) Locations of each of the detected motifs in the psbA upstream regions.

**Calculation of the energy requirement for S-PM2d development**

A conservative list of structural components of the S-PM2d virion comes from a proteomic investigation in (*1*) and are listed in Table S3. The mol equivalent requirement of ATP for polymerisation of pre-formed amino acids into these structural components was calculated based on 4 ATP equivalents being used for polymerisation of one amino acid into a growing polypeptide chain by ribosome activity as described in Stouthamer (1973). This is based on an average burst size of 10. The number of ATP molecules required to synthesise each amino acid *de* novo from oxidation of glucose is shown in Table S4.

Computing the ATP requirement for phage DNA replication is made difficult due to the uncertainty of the source of dNTPs. If one assumes that every dNTP of the S-PM2d genome is synthesised *de novo*, according to the predicted purine and pyrimidine biosynthetic pathways of *Synechococcus* sp. WH7803 shown in Fig. S2, it can be calculated that the mol ATP requirement for dNTP synthesis is 2.2 x 10^-17^ mol ATP. This is again based on a burst size of 10 and computed according to the exact dNTP frequency of the S-PM2d genome. However, given that a potential source of dNTPs is host chromosomal DNA/RNA and free DNA/RNA, the ATP requirement is expected to be much less. Cyanophage S-PM2d encodes multiple enzymes involved in nucleotide metabolism. For example an exonuclease (EBI Acc. No. CFW42411.1), a ribonucleotide reductase A (CFW42340.1), a ribonucleotide reductase B (CFW42343.1) and a thymidylate synthetase (CFW42406.1). Therefore, estimation of the ATP requirement for genome replication is probably significantly less than described above.

Table S3: Summary of the major structural components of the S-PM2d virion as reported in (*1*). The copy number is based on comparison to T4, or where homology to T4 is absent the copy number is conservatively assumed to be one. Four mol of ATP is required to add one amino acid to a growing polypeptide chain according to (*2*).

| Protein | S-PM2 ORF | Predicted copy number | Amino acid length | ATP molecules required for polymerisation | mol ATP required per virion (x10^-20^) |
| --- | --- | --- | --- | --- | --- |
| Gp6 | 83 | 12 | 602 | 28896 | 4.80 |
| hyp | 85 | 1 | 175 | 700 | 0.12 |
| Gp8 | 86 | 12 | 634 | 30432 | 5.05 |
| hyp | 89 | 1 | 1251 | 5004 | 0.83 |
| hyp | 90 | 1 | 168 | 672 | 0.11 |
| hyp | 92 | 1 | 306 | 1224 | 0.20 |
| hyp | 93 | 1 | 327 | 1308 | 0.22 |
| hyp | 94 | 1 | 379 | 1516 | 0.25 |
| Gp14 | 97 | 10 | 292 | 11680 | 1.94 |
| Gp15 | 98 | 6 | 266 | 6384 | 1.06 |
| Gp18 | 105 | 138 | 743 | 410136 | 68.10 |
| Gp19 | 106 | 144 | 204 | 117504 | 19.51 |
| Gp20 | 107 | 12 | 564 | 27072 | 4.50 |
| Gp22 | 110 | 115 | 392 | 180320 | 29.94 |
| Gp23 | 111 | 960 | 468 | 1797120 | 298.42 |
| Gp3 | 113 | 6 | 169 | 4056 | 0.67 |
| hyp | 148 | 1 | 316 | 1264 | 0.21 |
| hyp | 175 | 1 | 1095 | 4380 | 0.73 |
| hyp | 177 | 1 | 1177 | 4708 | 0.78 |
| Gp48 | 203 | 6 | 332 | 7968 | 1.32 |
| hyp | 222 | 1 | 295 | 1180 | 0.20 |
| hyp | 224 | 1 | 560 | 2240 | 0.37 |
| hyp | 226 | 1 | 1037 | 4148 | 0.69 |

Table S4: Amino acid frequency in virion structural proteins of cyanophage S-PM2d and theoretical ATP requirements of synthesis. Theoretical ATP requirements are from glucose and are reported in (*2*).

| Amino Acid | Symbol | Frequency of amino acids in structural proteins | ATP Requirement for synthesis of 1 mol of amino acid | ATP molecules required for *de novo* synthesis | mol of ATP required per virion (x10^20^) |
| --- | --- | --- | --- | --- | --- |
| Alanine | A | 767 | 1 | 767 | 0.13 |
| Arginine | R | 424 | -3 | -1272 | -0.21 |
| Asparagine | N | 856 | -2 | -1712 | -0.28 |
| Aspartic Acid | D | 734 | 0 | 0 | 0.00 |
| Cysteine | C | 34 | -3 | -102 | -0.02 |
| Glutamic Acid | E | 610 | 1 | 610 | 0.10 |
| Glutamine | Q | 365 | 0 | 0 | 0.00 |
| Glycine | G | 1120 | 0 | 0 | 0.00 |
| Histidine | H | 85 | -7 | -595 | -0.10 |
| Isoleucine | I | 833 | -1 | -833 | -0.14 |
| Leucine | L | 759 | 3 | 2277 | 0.38 |
| Lysine | K | 414 | 0 | 0 | 0.00 |
| Methionine | M | 137 | -4 | -548 | -0.09 |
| Phenylalanine | F | 523 | -2 | -1046 | -0.17 |
| Proline | P | 482 | 0 | 0 | 0.00 |
| Serine | S | 1086 | 0 | 0 | 0.00 |
| Threonine | T | 1107 | -2 | -2214 | -0.37 |
| Tryptophan | W | 106 | -5 | -530 | -0.09 |
| Tyrosine | Y | 472 | -2 | -944 | -0.16 |
| Valine | V | 838 | 2 | 1676 | 0.28 |


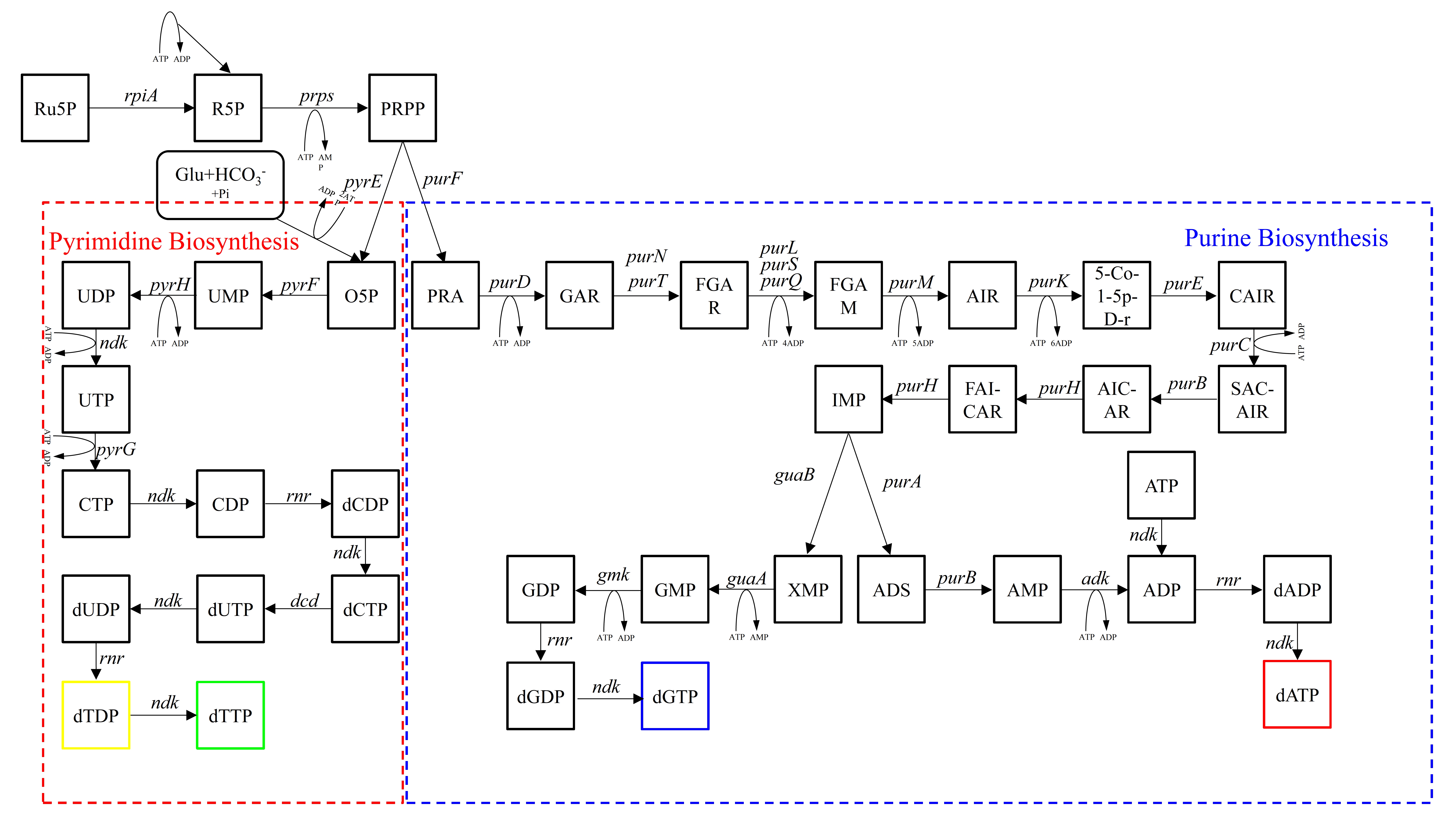


Figure S2: Predicted purine and pyrimidine biosynthetic pathways in Synechococcus sp. WH7803, used to calculate the theoretical ATP requirement of de novo dNTP biosynthesis for phage genome replication. Prediction of the biosynthetic pathways are based on the gene content of *Synechococcus* WH7803 and known biosynthetic pathways as reported in the KEGG database. Substrates: Ru5P= Ribulose-5-phosphate, R5P= Ribose 5-phosphate, PRPP= Phosphoribosyl pyrophosphate, O5P= Orotidine 5'-phosphate, UMP= Uridine monophosphate, UDP= Uridine diphosphate, UTP= Uridine triphosphate, CTP= Cytidine triphosphate, CDP= Cytidine diphosphate, dCDP= Deoxycytidine diphosphate, dCTP= Deoxycytidine triphosphate, dUTP= Deoxyuridine triphosphate, dUDP= Deoxyuridine diphosphate, dTDP= Deoxythymidine diphosphate, dTTP= Deoxythymidine triphosphate, PRA= Phosphoribosylamine, GAR= Glycineamide ribonucleotide, FGAR= Phosphoribosyl-N-formylglycineamide, FGAM= 5'-Phosphoribosylformylglycinamidine, AIR= 5-Aminoimidazole ribotide, 5-Co-1-5p-D-r= 5-Carboxyamino-1-(5-phospho-D-ribosyl)imidazole, CAIR= 5'-Phosphoribosyl-4-carboxy-5-aminoimidazole, SACAIR= Phosphoribosylaminoimidazolesuccinocarboxamide, AICAR= 5-Aminoimidazole-4-carboxamide ribonucleotide, FAICAR= 5-Formamidoimidazole-4-carboxamide ribotide, IMP= Inosine monophosphate, XMP= Xanthine monophosphate, GMP= Guanosine monophosphate, GDP= Guanosine diphosphate, dGDP= Deoxyguanosine diphosphate, dGTP= Deoxyguanosine triphosphate, ADS= Adenylosuccinate, AMP= Adenosine monophosphate, ADP= Adenosine diphosphate, ATP= Adenosine triphosphate, dADP= Deoxyadenosine diphosphate, dATP= Deoxyadenosine triphosphate. Genes: *rpiA*= Ribose-5-phosphate isomerase, *prps*= Phosphoribosyl pyrophosphate synthetase, *pyrE*= Orotate phosphoribosyltransferase, *pyrF*= Orotidine-5'-phosphate decarboxylase, *pyrH*= Uridylate kinase, *ndk*= Nucleoside diphosphate kinase, *pyrG*= CTP synthetase, *rnr*= ribonucleotide reductase, *dcd*= dCTP deaminase, *purF*= Amidophosphoribosyltransferase, *purD*= Phosphoribosylglycinamide synthetase, *purN*= Phosphoribosylglycinamide formyltransferase, *purT*= Glycinamide ribonucleotide transformylase, *purL*= Phosphoribosylformylglycinamide synthase subunit, *purS*= Phosphoribosylformylglycinamidine synthetase subunit, *purQ*= Phosphoribosylformylglycinamidine synthase subunit, *purM*= Phosphoribosyl-aminoimidazole synthase, *purK*= N5-carboxyaminoimidazole ribonucleotide synthase, *purE*= N5-carboxyaminoimidazole ribonucleotide mutase, *purC*= Phosphoribosyl-aminoimidazole-succinocarboxamide synthase, *purB*= Phosphoribosyl-aminoimidazole-succinocarboxamide synthase, *purH*= Phosphoribosylaminoimidazolecarboxamide formyltransferase, *guaB*= Inosine-5'-monophosphate dehydrogenase, *purA*= Adenylosuccinate synthase, *guaA*= GMP synthase, *gmk*= Guanylate kinase, *purB*= Adenylosuccinate lyase, *adk*= Adenylate kinase.


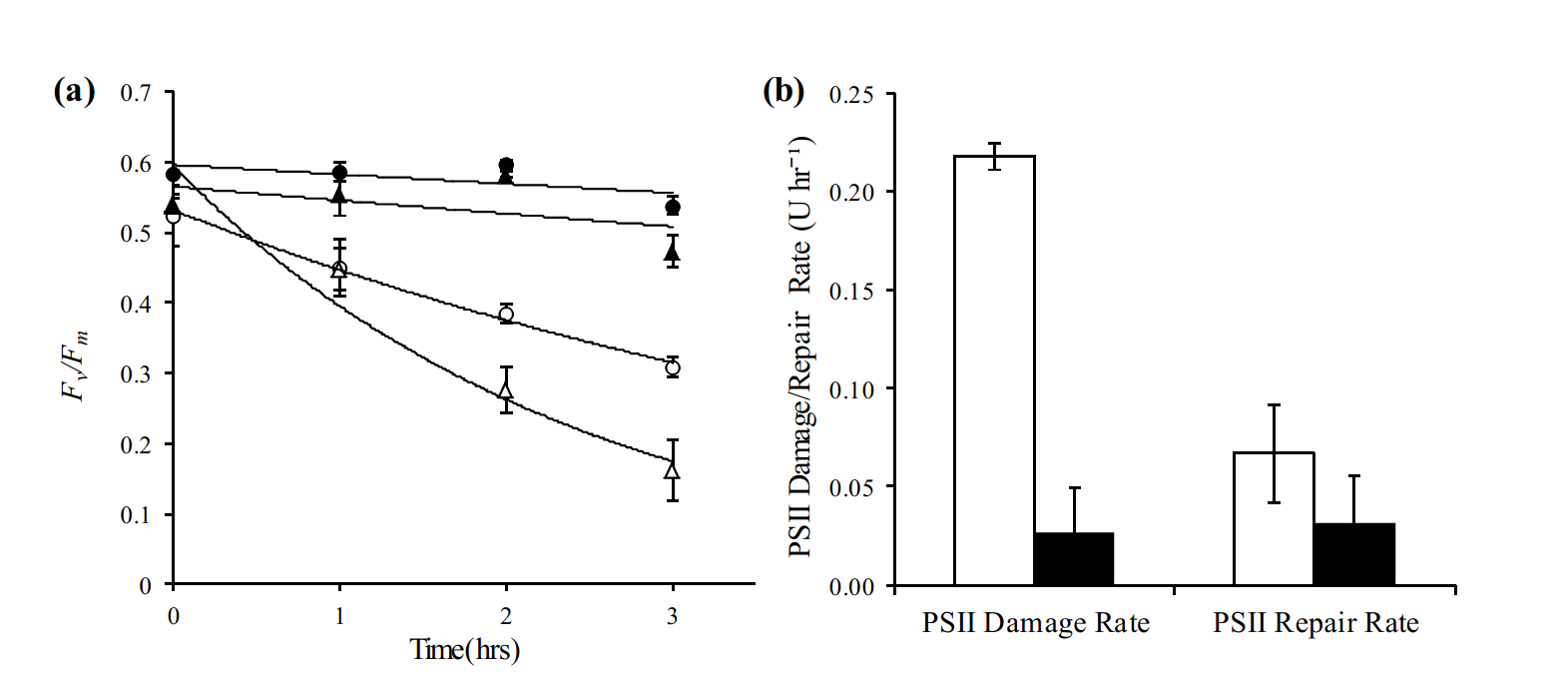


Figure S3: Demonstration of photoinhibition at HL treatment. (a) PSII maximum photochemical yield following addition of lincomycin. Black and white symbols indicate LL and HL respectively. Trianges indicate addition of lincomycin and circles are the control. (b) Calculated PSII damage and repair rates. Estimates are derived from the slope of the curves fitted through the data in (a) following (18). Black bars indicate LL and white bars indicate HL.


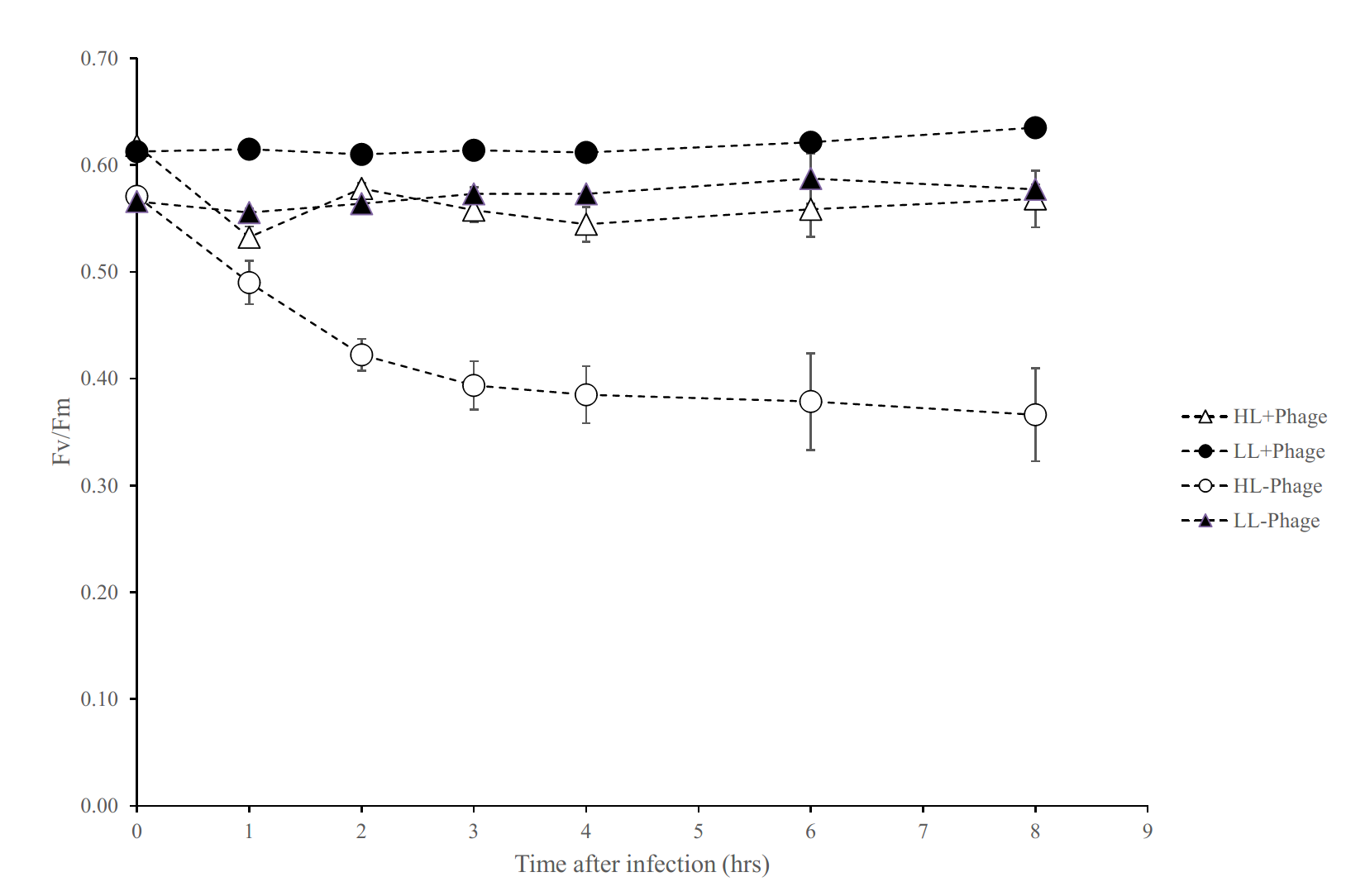


Figure S4: Raw Fv/Fm values over the infection period.

**References**

1. M. R. J. Clokie *et al.*, A proteomic approach to the identification of the major virion structural proteins of the marine cyanomyovirus S-PM2. *Microbiology*. **154**, 1775–82 (2008).

2. A. H. Stouthamer, A theoretical study on the amount of ATP required for synthesis of microbial cell material. *Antonie Van Leeuwenhoek*. **39**, 545–65 (1973).
