# Supplementary material for: Energy limitation of cyanophage development: implications for marine carbon cycling
Source: ISME J. 2018 Jan 29;12(5):1273–86. doi: 10.1038/s41396-017-0043-3 (PMC5931967; doi:10.1038/s41396-017-0043-3)
Supplement: Supplementary file 2 — Figure S1 [file 41396_2017_43_MOESM2_ESM.pdf]

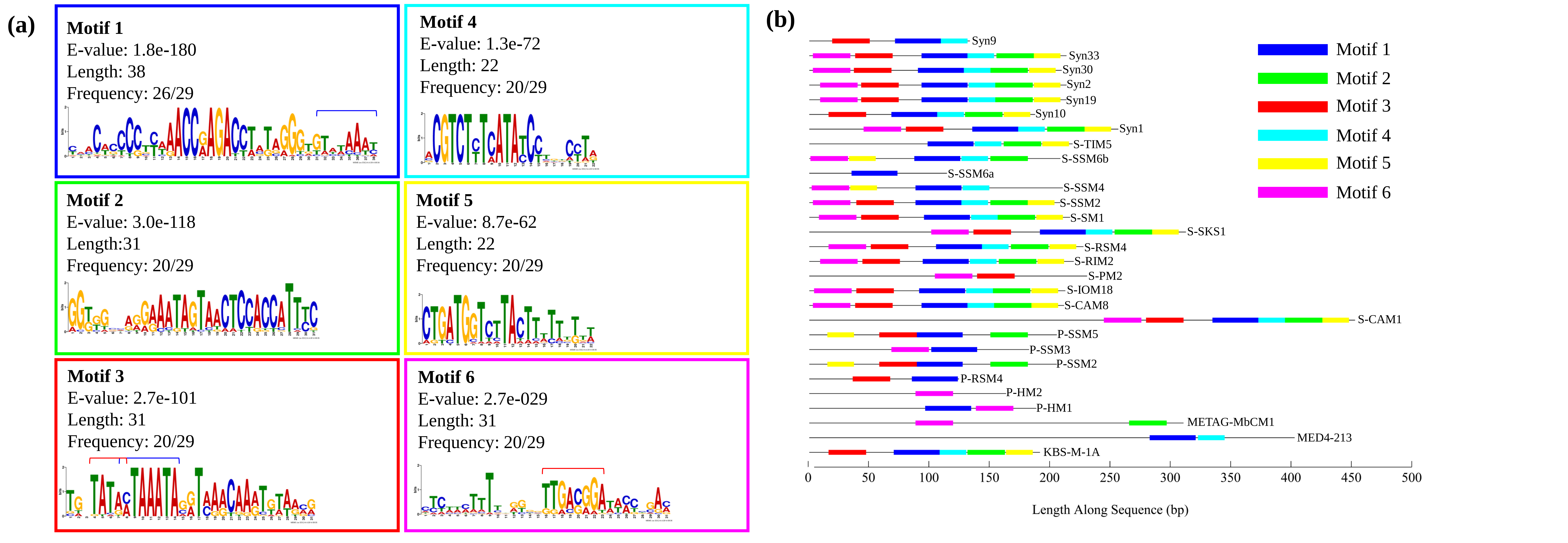
(a)
Motif 1
E-value: 1.8e-180
Length: 38
Frequency: 26/29
Motif 2
E-value: 3.0e-118
Length:31
Frequency: 20/29
Motif 3
E-value: 2.7e-101
Length: 31
Frequency: 20/29
Motif 4
E-value: 1.3e-72
Length: 22
Frequency: 20/29
Motif 5
E-value: 8.7e-62
Length: 22
Frequency: 20/29
Motif 6
E-value: 2.7e-029
Length: 31
Frequency: 20/29
(b)
Syn9
Syn33
Syn30
Syn2
Syn19
Syn10
Syn1
S-TIM5
S-SSM6b
S-SSM6a
S-SSM4
S-SSM2
S-SM1
S-SKS1
S-RSM4
S-RIM2
S-PM2
S-IOM18
S-CAM8
S-CAM1
P-SSM5
P-SSM3
P-SSM2
P-RSM4
P-HM2
P-HM1
METAG-MbCM1
MED4-213
KBS-M-1A
Motif 1
Motif 2
Motif 3
Motif 4
Motif 5
Motif 6
0
50
100
150
200
250
300
350
400
450
500
Length Along Sequence (bp)
